# New mutation of the ceruloplasmin gene in the case of a neurologically asymptomatic patient with microcytic anaemia, obesity and supposed Wilson’s disease

**DOI:** 10.1186/s12876-020-01237-8

**Published:** 2020-04-07

**Authors:** Mária Ondrejkovičová, Sylvia Dražilová, Monika Drakulová, Juan López Siles, Renáta Zemjarová Mezenská, Petra Jungová, Martin Fabián, Boris Rychlý, Miroslav Žigrai

**Affiliations:** 1 Department of Gastroenterology, Faculty of Medicine, University Hospital, Slovak Medical University, Bratislava, Slovakia; 2 Department of Internal medicine, Hospital Poprad, Poprad, Slovakia; 3 Hematologic Outpatient Clinic, Synlab Slovakia, s.r.o., Bratislava, Slovakia; 4 Imegen, Genetaq, Centro de Genetica Molecular, Malaga, Spain; 5 Laboratory of Medical Genetics, Alpha Medical s.r.o., Bratislava, Slovakia; 6 Department of Clinical Genetics, University Hospital, Bratislava, Slovakia; 7 Dr. Magnet, Department of Magnetic Resonance Imaging (MRI), Bratislava, Slovakia; 8 Cytopathos, Bratislava, Slovakia; 9 1st Department of Internal Medicine, Faculty of Medicine, University Hospital, Slovak Medical University, Limbova 5, 833 05 Bratislava, Slovakia

**Keywords:** Aceruloplasminemia, Ceruloplasmin, Wilson’s disease, Microcytic hypochrome anaemia, Case report

## Abstract

**Background:**

Aceruloplasminaemia is a very rare autosomal recessive disorder caused by a mutation in the ceruloplasmin gene, which is clinically manifested by damage to the nervous system and retinal degeneration. This classical clinical picture can be preceded by diabetes mellitus and microcytic anaemia, which are considered to be early manifestations of aceruloplasminaemia.

**Case presentation:**

In our report, we describe the case of a patient with aceruloplasminaemia detected in an early stage (without clinical symptoms of damage to the nervous system) during the search for the cause of hepatopathy with very low values of serum ceruloplasmin.

Molecular genetic examination of the CP gene for ceruloplasmin identified a new variant c.1664G > A (p.Gly555Glu) in the homozygous state, which has not been published in the literature or population frequency databases to date. Throughout the 21-month duration of chelatase treatment, the patient, who is 43 years old, continues to be without neurological and psychiatric symptomatology. We observed a decrease in the serum concentration of ferritin without a reduction in iron deposits in the brain on magnetic resonance imaging.

**Conclusion:**

Currently, there is no unequivocal recommendation of an effective treatment for aceruloplasminaemia. Early diagnosis is important in the neurologically asymptomatic stage.

## Background

Aceruloplasminaemia is a rare autosomal recessive disorder caused by a mutation in the ceruloplasmin gene linked to the accumulation of iron in an organism, mainly in the liver, brain, retina and pancreas. It is clinically manifested by retinal degeneration, diabetes mellitus, microcytic anaemia and neurological symptomatology [1]. The diagnosis of aceruloplasminaemia is often not confirmed before the origin of irreversible neurological symptomatology.

In our report, we describe the case of a patient with aceruloplasminaemia, which was identified in an early, neurologically asymptomatic stage while searching for the cause of chronic hepatopathy with low values of serum ceruloplasmin with originally supposed Wilson’s disease.

## Case presentation

A 41-year-old patient with several years of anamnesis of microcytic hypochromic anaemia after recurring miscarriages was initially examined in a district hepatology outpatient clinic because of slightly increased values of ALT activity. The physical examination was, apart from obesity (body mass index 30), in a normal range. Ultrasonographic examination revealed hyperechogenicity of the liver; therefore, we assumed the presence of liver steatosis. The basic laboratory examination confirmed microcytic hypochromic anaemia of a light degree. Virus and autoimmune hepatitis were excluded. The ceruloplasmin (CP) level was measured by the immune nephelometry assay in a BN II and BN ProSpec system (Siemens): human serum was mixed with anti-human CP immunoglobulin fraction, and the resulting immune complexes were measured by immune nephelometry (immune complexes can scatter the light; the source of incident light is the infrared high performance LED, and the scattered light intensity at a fixed angle of 13–24 degrees is measured by a photodiode detector) [2]. Regarding the low level of immunoreactive CP (measured by immune nephelometry) concentration (0.02 g/l), Wilson’s disease was suspected. The patient was sent to our outpatient clinic for the purpose of diagnostic final solution and treatment.

In the laboratory, hyposideraemia with paradoxically increased values of serum ferritin was found in addition to the abovementioned slightly increased ALT activity and microcytic hypochromic anaemia (complete laboratory and histological findings in the present patient at first examination are available in Table 1). The absence of Kayser-Fleischer ring, normal values of excretion of copper in urine (value: 346 nmol/24 h), genetic examination (including complete sequence analysis of the ATP7B chromosomal gene) and normal concentration of copper in dry matter of the liver (33 μg copper/g of dry liver tissue) excluded the supposed diagnosis of Wilson’s disease.

**Table 1:** Laboratory and histological findings in the present patient at first examination and after treatment

Biochemical variables	Results A	Results B	Results C	Normal values
AST, ukat/l	0,36	0,39	0,3	0,1–0,6
ALT, ukat/l	**0,66**	0,5	0,22	0,1 - 0,6
ALP, ukat/l	0,9	0,5	0.94	0,5–2,0
GGT, ukat/l	0,52	0,86	0,31	0,1–1,3
Glycemia, mmol/l	5,5	5,4	5,1	4,0 - 5,8
Ceruloplasmin (CP) concentration, g/l	**< 0,02**	**< 0,02**	**< 0,02**	0,15–0,3
u-Cu/24 h, nmol/24 h	346			< 1000
S-Cu, umol/l	**4,7**	**2,0**	**2,1**	12–24
S-Fe, umol/l	**3,42**	**4,2**	**5,3**	10,7–32
Ferritin, ng/ml	**675**	**461,39**	204	4,6–204
Saturation of transferrin, %	**4,9**	**7,0**	**7,3**	20–45
TG, mmol/l	1,53	1,06	1,38	**<** 1,7
Cholesterol, mmol/l	**5,19**	**5,62**	**5,9**	2,9 - 5,0
LDL, mmol/l	**3,68**	**3,67**	**3,83**	< 2,6
Albumin, g/l	47,6	42,8	45	35–52
**Imunology and virology**				
Autoantibodies (ANA, ANCA, a-LKM, AMA, ASMA)	negative			
Serology (HAV,HBV,HCV, HEV)	negative			
**BMI**	30	30	30	20–25
**Histology**				**Normal values**
ug cooper/gram of dry liver tissue	33			25–50
ug iron/gram of dry liver tissue	14,602			< 1600 μg/g

*AST* aspartate aminotransferase, *ALT* alanine aminotransferase, *ALP* alkaline phosphatase, *GGT* gamma-glutamyltransferase, u-urine, *s* serum, *Cu* cooper, *Fe* iron, *TG* triglycerides, *LDL* low density lipoprotein, *ANA* Anti-nuclear antibodies (Ab), *ANCA* Anti-neutrofil cytoplasm Ab, *a-LKM* Anti-liver/ kidney microsomal Ab, *AMA* Anti-mitochondrial Ab, *ASMA* Anti-smooth muscle Ab, *HAV* hepatitis A virus, *HBV* hepatitis B virus, *HCV* hepatitis C virus, *HEV* hepatitis E virus, *BMI* body mass index

**Results A** - first examination, **Results B** - 12 months after starting treatment, **Results C** - last examination (21 months after starting treatment)

**Comments**: hepatic enzyme levels (ALT, ALP, and GGT) and albumin were normal throughout the follow-up, the AST value was slightly increased before treatment, CP and serum copper were very low throughout the follow-up, low serum iron and transferrin saturation persisted, the ferritin level normalized during treatment, the patient had slightly elevated cholesterol and LDL (with normal values of TG) throughout the follow-up (without hypolipidaemic treatment), blood glucose values were normal, and weight and BMI remained unchanged

**Fig. 1:**
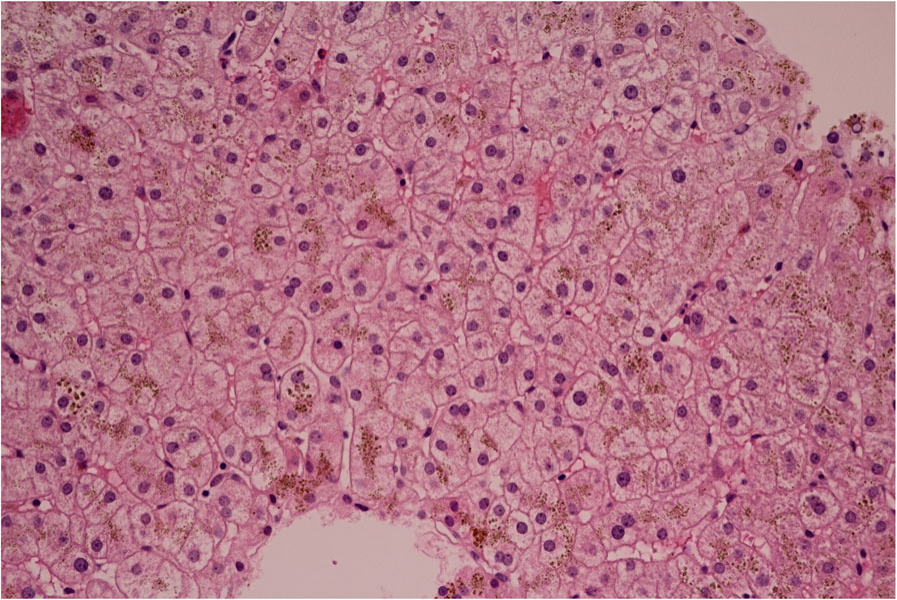
Histology of the liver. Haematoxylin-eosin staining. Almost no lobular inflammation, without steatosis and without fibrosis. 400x

**Fig. 2:**
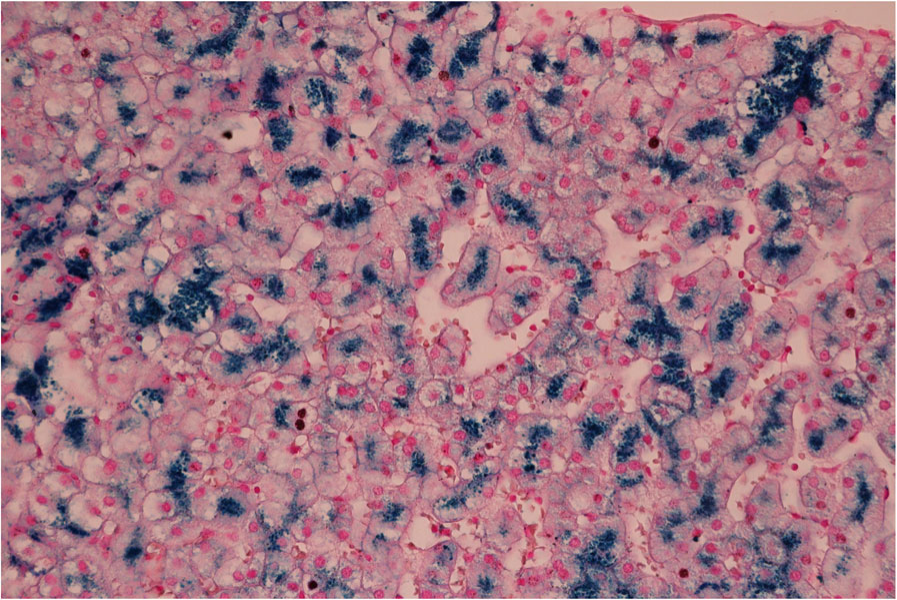
Liver histology. Perl’s stain. Strong positivity for parenchymatous iron. 600x

Histological examination of the liver revealed minimum lobular inflammation without fibrosis. The presence of liver steatosis was not confirmed (Fig. 1). Strong positivity of parenchymatous iron (Fig. 2) was a dominant finding. The accumulation of iron was confirmed by its quantitative determination (14,602 μg of iron/g of dry liver tissue, where the standard is up to 1600 μg/g of dry tissue).

Mutations were not found by extended genetic examination of hereditary haemochromatosis (STRIP method, 12 most frequent mutations in the HFE gene and 4 most frequent mutations in the TRF2 and FPN1 genes) and genetic examination of aceruloplasminaemia (two most frequent mutations of 125insTACAC and W858X).

The CP gene was sequenced by next-generation sequencing (MiSeq), which revealed a new mutation in the ceruloplasmin gene c.1664 > A (p.Gly555Glu).

**Fig. 3:**
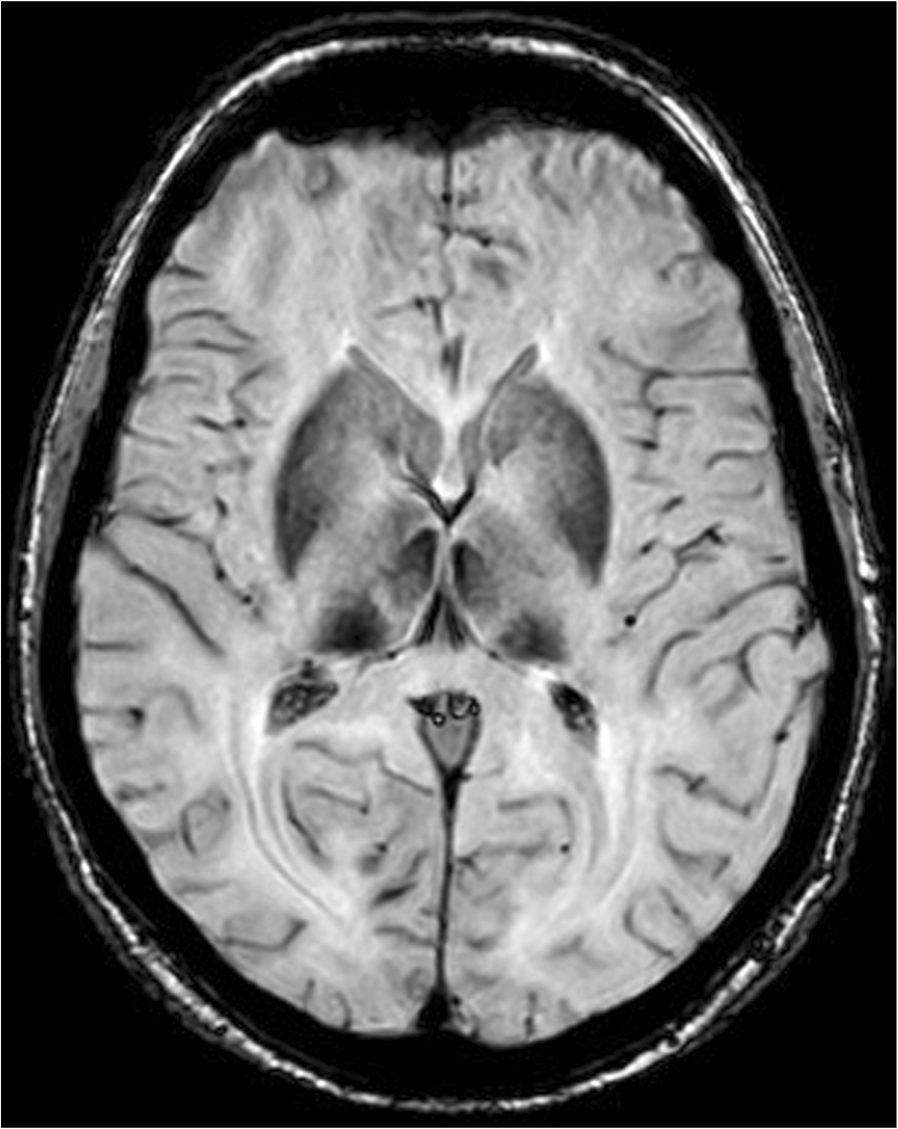
MRI of the brain. Susceptibility weighted imaging. Hypo-intensive changes in the basal ganglia and thalamus caused by deposits of iron

Although all in silico prediction tools (SIFT: Deleterious; Polyphen 2: Probably damaging; LTR: Deleterious; Mutation Taster: Disease causing; Mutation Assessor: High risk; FATHMM: Deleterious; SVM: Deleterious; LR: Deleterious) show deleterious effects, this change affects a conserved domain (the third cupredoxin domain of ceruloplasmin). MRI of the brain revealed excessive deposition of iron in the area of the basal ganglia, thalamus, mesencephalic nuclei and nucleus dentatus bilaterally (Fig. 3). The first neurological examination (and repeatedly during the follow-up) did not show any typical manifestation of the disease (ataxia, involuntary movements, cognitive dysfunction, Parkinsonism). Ophthalmological examination (including fundoscopy and visual field tests) didn’tn show retinal degeneration. Her visual acuity was not disturbed during the follow-up.

Our patient had normal fasting blood glucose values after repeated examinations, and an oral glucose tolerance test did not confirm the diagnosis of diabetes mellitus. The HOMA index was not calculated.

Chelatase treatment with deferasirox at a dose of 350 mg once daily during the first 9 months was started, and subsequently, from the 10th month of treatment, the dose was changed to 700 mg once daily. We recorded a progressive reduction in the serum concentration of ferritin in the laboratory. The patient continues to be without neurological and psychiatric symptomatology during the 21 months of chelatase treatment. Table 2 shows the dynamics of laboratory parameters during treatment. Control MRI (after 21 months of treatment) did not show changes in the iron concentration in the brain. During the indicated observation, no neuropsychiatric symptomatology was found.

**Table 2:** Laboratory parameters before the beginning and during the chelatase treatment

	12.12.2017	8.1.2018	8.2.2018	11.6.2018	4.9.2018	15.1.2019	28.5.2019
Serum iron- (μmol/l)	2,8	3,5	4	4,6	3,4	3,5	4
Total iron-binding capacity (μmol/l)	66	63,6	59,4	66,7	62,8	67,8	72,8
Transferrin saturation (%)	4,2	5,5	6,7	6,9	5,4	5,2	5,5
Unsaturated iron binding capacity (μmol/l)	63,2	60,1	55,4	62,1	59,4	64,3	68,8
Serum ferritin (μg/l)	519,38	394,42	342,35	348,16	362,32	331,01	192,18
Hemoglobin (g/l)	110	110	109	110	104	109	106
Mean corpuscular volume (fl)	77,1	76,8	77,3	77,4	77,8	74,7	75,9
Mean corpuscular hemoglobin (pg)	24,4	24,1	23,7	24,3	23,9	22,9	23,4

## Discussion and conclusions

Ceruloplasmin is a polypeptide composed of 1046 amino acid residues, which is encoded by a gene on the 3rd chromosome [3, 4].

In humans, ceruloplasmin occurs in two isoforms, which arise by alternative RNA “splicing” [5].

The soluble (secretory) form is expressed only in hepatocytes [6].

It constitutes the main transporter of copper in the plasma, has antioxidant properties and participates in the homeostasis of nitric oxide (NO) [6, 7].

The membrane-bound form of ceruloplasmin, associated with glycosylphosphatidylinositol (GPI-ceruloplasmin), is expressed in astrocytes, Sertoli cells, retinal cells, lungs, liver, kidneys, macrophages and immune system cells [8, 9].

GPI-ceruloplasmin participates in maintaining iron homeostasis in the brain. Under physiological conditions, iron is continuously transferred between astrocytes and neurons by means of transferrin, which acts as a pendulum. GPI-ceruloplasmin oxidizes Fe^2+^ on astrocyte protrusions, enabling binding and transport of Fe^3+^ to transferrin to meet the needs of neurons. When GPI-ceruloplasmin is absent, astrocytes are not capable of transporting iron through transferrin to neurons, which is why they catch it from alternative sources (citrate, ascorbate). The surplus of iron in astrocytes leads to oxidizing damage and a reduction in protective function in relation to neurons [1, 7].

Serum CP may be decreased in conditions with marked renal or enteric protein loss, malabsorption syndromes or severe end-stage liver disease and fulminant liver failure as a result of decreased hepatic protein synthesis [10].

An association between the low concentration and activity of CP and non-alcoholic fatty liver disease (NAFLD) with a high NAFLD score (NAS ≥ 5) in children was reported by Nobili et al. CP was associated with the odds of NASH, ballooning, inflammation and steatosis [11].

An inherent reduction in CP is typical of Wilson’s disease (due to mutations in the ATP7B gene, which encodes a copper-transporting P-type ATPase, with subsequent accumulation of copper in affected tissues) and of Menkes disease (due to mutations in the ATB7A gene causing a disorder of intestinal copper uptake) [10]. TMEM199-CDG is a rare genetic liver disease with abnormal glycosylation, chronically elevated serum transaminases, steatosis and low serum ceruloplasmin [12, 13].

In the present case, we repeatedly detected very low serum ceruloplasmin values in a patient with obesity. However, the patient had no arterial hypertension, impaired glucose tolerance or elevation of triacylglycerol (typical of NAFLD). Ultrasonographic examination of the liver revealed increased liver echogenicity. However, histological examination did not confirm liver steatosis, hepatocyte ballooning or fibrosis, and copper accumulation in the liver (typical of Wilson’s disease) was also excluded. Genetic examination revealed a mutation in the CP gene.

**Aceruloplasminaemia** is a rare, autosomal recessive hereditary disorder caused by mutations of the ceruloplasmin gene. The prevalence is estimated to be 1/2,000,000 in the Japanese population [14]. The prevalence in other parts of the world is not known.

**Clinical manifestations** of aceruloplasminaemia include retinal degeneration, diabetes mellitus, microcytic anaemia and neuropsychiatric symptomatology (cerebellar ataxia, dysarthria, nystagmus, dystonia, tremor, chorea, rigidity, akinesia, behaviour malfunctions, cognitive deficit, dementia) [14, 15].

Neuropsychiatric symptomatology mostly arises in the fifth life decade, and it can often be preceded by diabetes mellitus and microcytic anaemia, which are considered to be early manifestations of aceruloplasminaemia [16, 17].

The basic **laboratory finding** is a very low measurable concentration of ceruloplasmin in serum. Other laboratory findings include a low concentration of serum iron, low saturation of transferrin (10%), elevated concentration of serum ferritin and microcytic anaemia of light degree [18]. Visual examination is performed to diagnose peripheral degeneration of the retina [16]. MRI of the brain is used to find the accumulation of iron in the basal ganglia, thalamus, mesencephalon and cerebellum [19].

In the case of our patient, aceruloplasminaemia was not found before searching for the cause of chronic hepatopathy with low concentrations of serum ceruloplasmin. The initial supposed diagnosis of Wilson’s disease was ruled out by complex diagnostics. The finding of parenchymatous accumulation of iron in the liver led to the genetic examination focused on diseases causing accumulation of iron.

Extended genetic analysis revealed the variant c.1664G > A (p.Gly555Glu) of ceruloplasmin in the homozygous state. This mutation has not been previously described in the literature or population frequency databases and has unknown clinical significance according to the American College of Medical Genetics and Genomics standards [20, 21].

However, all in silico prediction tools showed this variant to be pathogenic or disease causing. Moreover, the 555 AA position in the CP protein belongs to the third cupredoxin domain of CP, a very important functional domain [22]. However, neither functional assays nor co-segregation of the variant in families have been performed to reclassify it as a likely pathogenic variant. We do not currently have a genetic examination of the patient’s relatives. Thus, we cannot determine the enzymatic or biological properties of altered CP and their relationship with the disease phenotype. We plan to add this to our investigation after obtaining informed consent.

Recently, there has not been a definite recommendation for aceruloplasminaemia treatment. Medical possibilities include chelatase substances (deferoxamine, deferasirox, deferiprone), vitamin E, vitamin C, zinc preparations, fresh frozen plasma and minocycline. Experience with these substances has been, however, limited to the results of individual case histories of patients [23].

Considering the most often used chelatase treatment, a reduction in serum ferritin and a reduction in the content of iron in the liver were observed. MRI mostly did not show changes in iron deposits of iron in the brain during chelatase treatment [24]. The changes in neurological symptomatology covered a wide range from improvement to stabilisation to deterioration [24, 25].

When the patient is without significant neurological symptomatology, ideally when they are asymptomatic, an early start to chelatase treatment is considered to be a basic factor for its success [17, 26]. In accordance with the literature data, we recorded a reduction in the concentration of serum ferritin. MRI did not show a reduction in the iron content in the brain.

Neurological symptomatology arises in patients with aceruloplasminaemia, usually in the fifth decade. Therefore, it has not yet been possible to assess the effect of treatment on its postponement.

## Data Availability

Patient documentation is available from the corresponding author on request.

## Abbreviations

ATPase 7B: Adenosine triphosphatase 7B
ATPase 7A: Adenosine triphosphatase 7A
ALT: Alanine aminotransferase
CDG: Congenital disorder of glycosylation
CP: Ceruloplasmin
GPI: Glycosylphosphatidylinositol
MRI: Magnetic resonance imaging
NO: Nitrogen oxide
NAFLD: Nonalcoholic fatty liver disease
NAS: Nonalcoholic fatty liver disease score
RNA: Ribonucleic acid
STRIP: STRucture In Populations
TRF2: Transferrin receptor 2
FPN1: Ferroportin-1

## References

[1] Miyajima H (2015). Aceruloplasminemia. Neuropathology.

[2] Stanley J (2002). Essentials of immunology and serology.

[3] Takahashi N, Ortel TL, Putnam FW (1984). Single-chain structure of human ceruloplasmin: the complete amino acid sequence of the whole molecule. Proc Natl Acad Sci U S A.

[4] Yang F, Naylor SL, Lum JB, Cutshaw S, McCombs JL, Naberhaus KH (1986). Characterization, mappiong, and expression of the human ceruloplasmin gene. Proc Natl Acad Sci U S A.

[5] Yang F, Friedrichs WE, Cupples RL, Bonifacio MJ, Sanford JA, Horton WA (1990). J Biol Chem.

[6] Hellman NE, Gitlin JD (2002). Ceruloplasmin metabolism and function. Annu Rev Nutr.

[7] Shiva S, Wang X, Ringwood LA, Xu X, Yuditskaya S, Annavajihala V (2006). Ceruloplasmis is a NO oxidase and nitrite synthase that determines endocrine NO homeostasis. Nat Chem Biol.

[8] Kono S, Yoshida K, Tomosugi N, Terada T, Hamaya Y, Kanaoka S (1802). Biological effects of mutant ceruloplasmin on hepicidin-mediated internalization of ferroportin. Biochim Biophys Acta.

[9] Musci G, Polticelli F, Bonaccorrsi di Patti MC (2014). Ceruloplasmin-ferroportin system of iron traffic in vertebrates. World J Biol Chem.

[10] Ferenci P (2012). EASL clinical practice guidelines: Wilson s disease. J Hepatol.

[11] Nobili V, Siotto M, Bedogni G (2013). Levels of serum ceruloplasmin associate with pediatric nonalcoholic fatty LIver disease. J Pediatr Gastroenterol Nutr.

[12] Vajro P (2018). Three unreported cases of TMEM199-CDG, a rare genetic liver disease with abnormal glycosylation. Orphanet J Rare Dis.

[13] Jansen JC, Timal S, van Scherpenzeel M (2016). TMEM199 deficiency is a disorder of golgi homeostasis characterized by elevated aminotransferases, alkaline phosphatase, and cholesterol and abnormal glycosylation. Am J Hum Genet.

[14] Miyajima H, Kohno S, Takahashi Y, Yonekawa O, Kanno T (1999). Estimation of the gene frequency of aceruloplasminemia in Japan. Neurology..

[15] Vroegindeweijl LH, Langendonk JG, Lengeveld M, Hoogendoorn M, Kievit JA, Do Raimondo D (2017). New insights in the neurological phenotype of aceruloplasminemia in Caucasian patients. Parkinsonism Relat Disord.

[16] Vroegindeweij LH, van der Beek EH, Boon AJ, Hoogendoorn M, Kievit JA, Wilson JH, et al. Aceruloplasminemia presents as type 1 diabetes in non-obese adults: a detailed case series. Diabet Med. 2015. 10.1111/dme.12712. 10.1111/dme.12712 25661792

[17] Pelucchi S, Mariani R, Ravasi G, Pelloni L, Marano M, Tremolizzo L (2018). Phenotypic heterogenity in seven Italian cases aceruloplasminemia. Parkinsonism Relat Disord.

[18] Marchi G, Busti F, Zidanes AL, Castagna A, Girelli D. Aceruloplasminemia: a severe neurodegenerative disorder deserving an early diagnosis. Front Neurosci. 2019. 10.3389/fnins.2019.00325. 10.3389/fnins.2019.00325 PMC6460567 31024241

[19] Parks NE, Vandorpe RA, Moeller JJ. Teaching neuroimages: neurodegeneration with brain iron accumulation in aceruloplasminemia. Neurology. 2013. 10.1212/01.wnl.0000435557.21319.ad. 10.1212/01.wnl.0000435557.21319.ad 24218322

[20] Stenson PD, Mort M, Ball EV, Shaw K, Philips A, Cooper DN (2014). The human gene mutation database: a building a comprehensive mutation repository for clinical and molecular genetics, diagnostic testing and personalized genomic medicine. Hum Gene Ther.

[21] Landrum MJ, Lee JM, Bensom M, Brown G, Chao C, Chitipiralla S (2016). ClinVar: public archive of interpretations of clinically relevant variants. Nucleic Acids Res.

[22] Apweiler R, Bairoch A, Wu CH, Barker WC, Boeckmann B, Ferro S (2004). UniProt: the universal protein knowledgebase. Nucleic Acids Res.

[23] Piperno A, Alessio M. Aceruloplasminemia: waiting for an efficient therapy. Front Neurosci. 2018. 10.3389/fnins.2018.00903. 10.3389/fnins.2018.00903 PMC6290325 30568573

[24] Finkenstedt A, Wolf E, Hofner E, Gasser BL, Bosch S, Bakry R (2010). Hepatic but not brain iron is rapidly chelated by deferasirox in aceruloplasminemia due to a novel gene mutation. J Hepatol.

[25] Skidmore FM, Drago V, Foster P, Schmalfuss IM, Heilman KM, Streiff RR (2008). Aceruloplasminemia with progressive atrophy without brain iron overload: treatment with oral chelation. J Neurol Neurosurg Psychiatry.

[26] Doyle A, Rusli F, Bhathal P. Aceruloplasminaemia: a rare but important cause of iron overload. BMJ Case Rep. 2015. 10.1136/bcr-2014-207541. 10.1136/bcr-2014-207541 PMC4434379 25976187

